# Haplotype CGC from XPD, hOGG1 and ITGA2 polymorphisms increases the risk of nasopharyngeal carcinoma in Malaysia

**DOI:** 10.1371/journal.pone.0187200

**Published:** 2017-11-09

**Authors:** Eng-Zhuan Ban, Munn-Sann Lye, Pei Pei Chong, Yoke-Yeow Yap, Siew Ying Crystale Lim, Hejar Abdul Rahman

**Affiliations:** 1 Department of Community Health, Faculty of Medicine and Health Sciences, Universiti Putra Malaysia, Serdang, Malaysia; 2 Department of Biomedical Science, Faculty of Medicine and Health Sciences, Universiti Putra Malaysia, Serdang, Malaysia; 3 Department of Otorhinolaryngology, Faculty of Medicine and Health Sciences, Universiti Putra Malaysia, Serdang, Malaysia; 4 Faculty of Applied Sciences, UCSI University, Cheras, Malaysia; Istituto di Genetica Molecolare, ITALY

## Abstract

**Background:**

8-oxoG, a common DNA lesion resulting from reactive oxygen species (ROS), has been shown to be associated with cancer initiation. hOGG1 DNA glycosylase is the primary enzyme responsible for excision of 8-oxoG through base excision repair (BER). Integrins are members of a family of cell surface receptors that mediate the cell-cell and extracellular matrix (ECM) interactions. Integrins are involved in almost every aspect of carcinogenesis, from cell differentiation, cell proliferation, metastasis to angiogenesis. Loss of ITGA2 expression was associated with enhanced tumor intravasation and metastasis of breast and colon cancer. XPD gene encodes DNA helicase enzyme that is involved in nucleotide excision repair (NER). It is shown in previous research that XPD homozygous wildtype Lys/Lys genotype was associated with higher odds of NPC.

**Methods:**

We conducted a 1 to N case-control study involving 300 nasopharyngeal carcinoma (NPC) cases and 533 controls matched by age, gender and ethnicity to investigate the effect of hOGG1 Ser326Cys, ITGA2 C807T and XPD Lys751Gln polymorphisms on NPC risk. Linkage disequilibrium and haplotype analysis were conducted to explore the association of allele combinations with NPC risk. Restriction fragment length polymorphism (RFLP-PCR) was used for DNA genotyping.

**Results:**

No significant association was observed between hOGG1 Ser326Cys and ITGA2 C807T polymorphisms with NPC risk after adjustment for age, gender, ethnicity, cigarette smoking, alcohol and salted fish consumption. Lys/Lys genotype of XPD Lys751Gln polymorphism was associated with increased NPC risk (OR = 1.60, 95% CI = 1.06–2.43). Subjects with history of smoking (OR = 1.81, 95% CI = 1.26–2.60), and salted fish consumption before age of 10 (OR = 1.77, 95% CI = 1.30–2.42) were observed to have increased odds of NPC. The odds of developing NPC of CGC haplotype was significantly higher compared to reference AGC haplotype (OR = 2.20, 95% CI = 1.06–4.58).

**Conclusion:**

The allele combination of CGC from hOGG1, ITGA2 and XPD polymorphisms was significantly associated with increased odds of NPC.

## Introduction

Nasopharyngeal carcinoma (NPC) develops commonly in the Fossa of Rosenmuller of the nasopharynx. It is a rare malignancy in most parts of the world with an annual frequency less than 1 per 100 000 population [1]. Certain populations such as Chinese living in Guangdong province of Mainland China and Southeast Asia as well as natives from Arctic region (Alaska and Greenland) experience a much higher NPC risk compared to the rest of the world [2]. NPC is the 4^th^ most common cancer in Malaysia in 2007 [3]. Given the increasing incidence of NPC cases and the fact that many cases are diagnosed at an advanced stage [4], it is important to find ways of ensuring early diagnosis and prompt treatment. This is challenging as the nasopharynx is not easily visualized and accessed. Discovering biomarkers for NPC screening is one of the ways in which a susceptible population could be identified early, which will help physicians in early detection and treatment of NPC.

Several environmental factors have been shown to be consistently associated with NPC. EBV infection [5], consumption of salted fish at an early age [6–7] (possibly due to nitrosamines mutagenicity), prolonged occupational exposure to wood dust [8] and long-term cigarette smoking [9] are examples of risk factors implicated in NPC carcinogenesis. In addition, normal cellular metabolic processes are also capable of producing hydroxyl radicals that can cause oxidative damage to DNA [10]. Oxidative stress has been linked to increased cancer risk via reactive oxygen species (ROS) acting in different stages of tumorigenesis [11]. One common mutagenic by-product resulting from oxidative damage is 8-oxo-7,8-dihydroguanine (8-oxoG), which is a G:C to T:A transversion causing agent [12]. Human 8-oxoguanine DNA glycosylase 1 (hOGG1) is the primary enzyme responsible for excision of 8-oxoG through base excision repair (BER). Short-patch BER removes 8-oxoG through the action of DNA glycosylase and AP lyase followed by the re-synthesis of DNA by DNA polymerase β. DNA is ligated by DNA ligase III eventually to complete the repair [13]. hOGG1 protein initiates BER via its ability to identify the damaged base. The presence and functionality of hOGG1 protein affects the level of BER activity directly. Several studies on association of hOGG1 Ser326Cys polymorphism with various cancers demonstrated that hOGG1-Cys326 conferred higher risk of cancer [14–17]. However, a study conducted in a Chinese population suggested otherwise with hOGG1-Ser326 conferring increased cancer risk instead [18]. Similarly for NPC, studies from different countries showed inconsistent results. Cho et al [19] demonstrated that Ser/Cys and Cys/Cys genotypes of hOGG1 gene (Ser326Cys) is associated with altered risk of NPC (OR = 1.6, 95% CI = 1.0–2.6). Laantri et al however showed that neither Ser/Cys nor Cys/Cys genotypes of hOGG1 gene (Ser326Cys) were significantly associated with NPC risk (OR = 1.22, 95% CI = 0.77–1.90) [20].

Xeroderma pigmentosum group D (XPD) gene encodes for 5’-3’ DNA helicase enzyme that is involved in transcription factor IIH (TFIIH) complex of nucleotide excision repair (NER) [21]. TFIIH is mainly made up of 2 sub-complexes which are the core and cdk-activating kinase (CAK) complex [22]. 6 sub-units namely XPB, p62, p52, p44, p34 and p8 combined to form the core whereas another 3 sub-units cdk7, cdk-activating kinase assembly factor I (MATI) and cyclin H bound to form CAK complex [22]. The remaining component XPD physically bridges 2 sub-complexes together to form a functional TFIIH complex [23–24]. TFIIH complex is responsible for the dual-incision process in NER that helps to unwind the DNA at the damaged region [25]. Deficient XPD-p44 interaction results in impaired unwinding of DNA in NER due to sub-optimal helicase activity [26]. XPD Lys751Gln polymorphism is located in carboxy terminal domain (CTD) where XPD-p44 interaction takes place [27]. XPD homozygous wildtype Lys/Lys genotype has been shown to be associated with higher odds of NPC (OR = 1.58, 95% CI = 1.05–2.38, p = 0.028) [28].

Integrins are members of a family of cell surface receptors that mediate the cell-cell and cell-extracellular matrices (ECM) interactions [29]. It has been demonstrated that integrins played an important role in apoptosis [29], tumor angiogenesis [30] and metastasis [31]. Integrins are heterodimeric and consist of 2 transmembrane glycoproteins (α and β) that are non-covalently bound together [32]. Thus far, there are 16 α and 8 β subunits in the integrin family that combine and produce more than 22 different αβ cell surface receptors [32]. Integrin α2 is an important collagen receptor that is mainly expressed on platelets and epithelial cells [33]. Under normal cell differentiation, expression of ITGA2 is regulated and kept within normal range but its over-expression is associated with decreased tumor cells motility and invasiveness [34–36]. Loss of ITGA2 in cancer cells is associated with metastasis in breast and colon carcinoma [33, 37]. ITGA2 C807T polymorphism is a silent nucleotide change in position 807 (TTC/TTT, rs1126643) which resulted in no amino acid change. Recent studies have indicated that ITGA2 C807T polymorphism was associated with increased risk of various cancers namely colorectal and breast carcinoma [38–39].

We describe results from a matched case-control study investigating the effect of hOGG1 Ser326Cys, ITGA2 C807T and XPD Lys751Glu polymorphisms on the risk of NPC.

## Materials and methods

The study was approved by the Medical Research Ethics Committee of the Ministry of Health Malaysia (NMRR-11-1038-10007). Written informed consent was obtained from all research participants involved in this study. We assumed the exposure rate of hOGG1 Ser326Cys polymorphism in controls at 61% [19] and estimated that this polymorphism could increase NPC risk by 100%. Using the formula adopted by Schlesselman [40] on matched case-control study, with two sided alpha level of 0.05, 196 matched pairs were needed to attain a power of 90% to detect a 100% increase in NPC risk in the proportion of patients with hOGG1 Ser326Cys polymorphism. 300 histologically confirmed NPC cases and 533 healthy controls were recruited from two public hospitals in this study. For NPC cases, the inclusion criteria were histologically confirmed NPC patients who were diagnosed from the year 2008 onwards. NPC cases who were 18 years of age and below at the time of recruitment were excluded. The inclusion criteria for healthy controls were individuals that have resided in Malaysia for at least 5 years and without having had a history of cancer. All controls were matched to the cases by age (±3 years), gender and ethnicity. Personal information on demographic factors, smoking status, alcohol and salted fish consumption were collected at recruitment. Smoking status and alcohol consumption were divided into 2 categories: never/ever smoked and never/ever consumed alcohol. For salted fish consumption, classification used was never/ever consumed salted fish at age of 10.

### DNA extraction and storage

2 ml of venous blood was obtained from every research subject. Fresh blood was immediately placed into an EDTA coated vacutainer. Filled EDTA tube was stored on ice and transferred back on the same day to the laboratory in the university to be processed. DNA was extracted from the blood using QIAamp^®^ DNA mini kit (QIAGEN, Venlo, Netherlands) and immediately stored in minus 20°C freezer until further use.

### DNA genotyping

**Fig 1:**
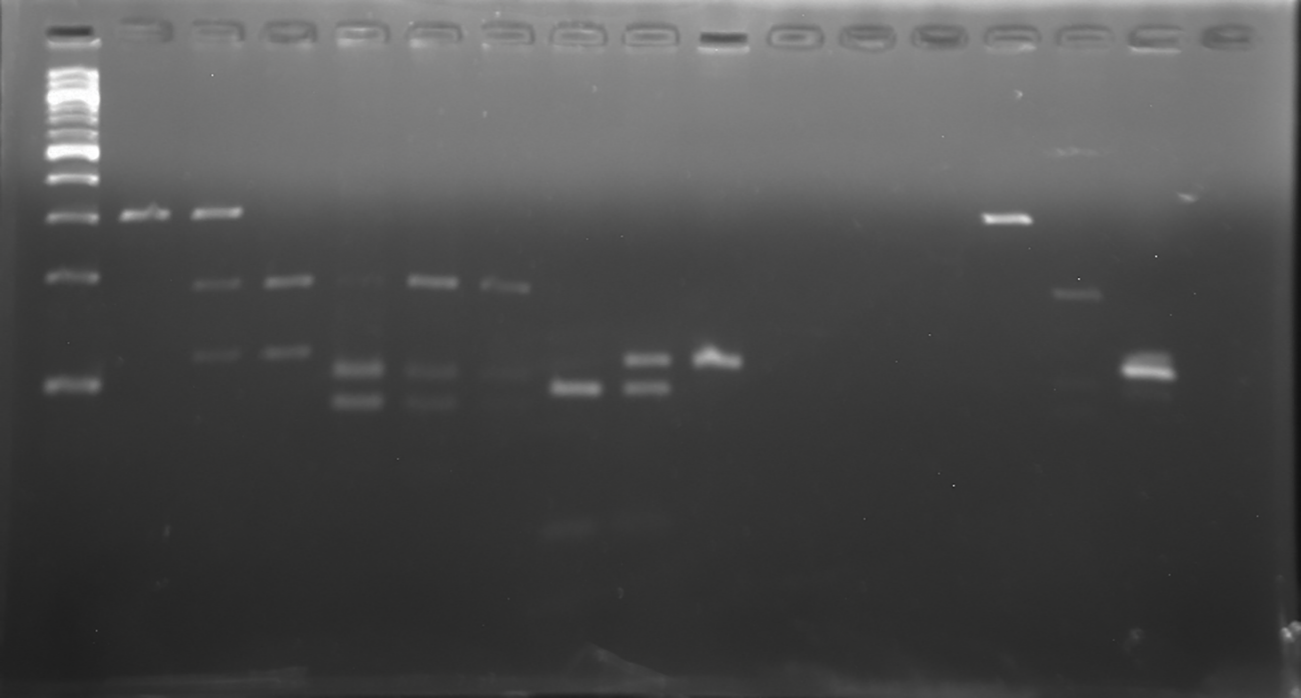
Gel electrophoresis of PCR-RFLP products for representative blood samples for the hOGG1 Ser326Cys polymorphism. Lane M represents 100bp DNA ladder marker (QIAgen), lanes 1 represents positive control (RFLP reaction with genotype-known PCR product), lanes 3 represents Ser/Ser genotype (302bp), lanes 2, 5, 6, 8 and 10–13 represent Ser/Cys genotype (302bp, 186bp and 116bp), lanes 4, 7 and 9 represent Cys/Cys genotype (186bp and 116bp), lane 14 represents negative controls (RFLP reaction without PCR product) and lane 15 represents negative control (RFLP reaction without PCR product but with restriction enzyme, *Fnu4H1*).

hOGG1 Ser326Cys (rs1052133), ITGA2 C807T (rs1126643) and XPD Lys751Gln (rs13181) polymorphisms were assessed by using RFLP-PCR (Restriction Fragment Length Polymorphism). Sequence of forward and reverse primers used in DNA genotyping are listed in Table 1. Outcome of the PCR for the XPD, hOGG1 and ITGA2 genotyping were products of 302 bp, 184 bp and 115 bp respectively. The details on PCR composition, PCR condition and RFLP digestion are listed in Table 2. For each polymorphism, there were 3 possible results depending on the subject’s genotype (Fig 1). In the case of XPD, samples were identified as homozygous Lys/Lys if the results showed full digestion with 102 bp and 82 bp product. Homozygous Gln/Gln samples showed only single PCR product that was 184 bp in size. All 3 products of different sizes were observed for heterozygous Lys/Gln. For hOGG1, homozygous Ser/Ser showed only a single 302bp product while homozygous Cys/Cys was fully digested into 2 different products that were 186 bp and 116 bp in size. All 3 products of different sizes were observed for heterozygous Ser/Cys. For ITGA2, homozygous CC was fully digested into 2 products that were 92 bp and 23 bp in size. For homozygous TT, no digestion occurred and only a single 115 bp product was visible. All 3 products of different sizes were observed for heterozygous CT. For quality control, 10% of the total PCR products were sent for DNA sequencing to confirm the results of RFLP-PCR.

**Table 1:** Sequence of forward and reverse primers used in DNA genotyping.

Polymorphisms	Forward primer sequence	Reverse primer sequence
hOGG1 (rs1052133)	5’-CTT CCA CCT CCC AAC ACT GTC AC-3’	5’-GTG CCT GGC CTT TGA GGT AGT C-3’
ITGA2 (rs1126643)	5’-GTG TTT AAC TTG AAC ACA TAT-3’	5’-ACC TTG CAT ATT GAA TTG CTT-3’
XPD (rs13181)	5′-CCC CCT CTC CCT TTC CTC TG-3′	5′-AAC CAG GGC CAG GCA AGA C-3′

**Table 2:** Details on PCR composition, PCR condition and RFLP digestion of XPD, hOGG1 and ITGA2 polymorphism.

Polymorphisms	XPD Lys751Gln (rs13181)	hOGG1 Ser326Cys (rs1052133)	ITGA2 C807T (rs1126643)
PCR composition	25μl PCR reaction consisting of 12.5μl of GoTaq^®^ Green Master Mix (Promega, USA), 0.5μl of each primer (from working concentration of 10μm), 0.5 μl of genomic DNA, and the remaining was nuclease free water.	25μl PCR reaction with 12.5μl of GoTaq^®^ Green Master Mix (Promega, USA), 0.5μl of each primer (from working concentration of 10μm), 0.5 μl of genomic DNA, and the remaining was nuclease free water.	25μl PCR reaction consisting of 12.5μl of GoTaq^®^ Green Master Mix (Promega, USA), 1.0μl of each primer (from working concentration of 10μm), 0.5 μl of genomic DNA, and the remaining was nuclease free water.
PCR condition	95°C for 5 minutes, 35 cycles each of 95°C for 40 seconds, followed by 56°C for 30s and 72°C for 30s, with final extension of 72°C for 5 min.	95°C for 5 minutes, 32 cycles each of 95°C for 30 seconds, followed by 63°C for 30s and 72°C for 30s, with final extension of 72°C for 5 min.	95°C for 5 minutes, 35 cycles each of 95°C for 30 seconds, followed by 55°C for 30s and 72°C for 30s, with final extension of 72°C for 5 min.
RFLP digestion	PCR product was digested by restriction enzyme MboII (New England Biolabs, Ipswich, MA, UK).	PCR product was digested by restriction enzyme Fnu4HI (New England Biolabs, Ipswich, MA, UK).	PCR product was digested by restriction enzyme TaqI (New England Biolabs, Ipswich, MA, UK).

### Statistical analysis

Relative frequencies were used to describe variables studied including socio-demographic and exposure data using SPSS version 21. Deviation from Hardy Weinberg equilibrium (HWE) was tested using Court Lab Calculator on controls [41]. Conditional logistic regression (STATA 10) was used to estimate adjusted odds ratio (ORs) and 95% confidence interval (CI) for NPC risk comparing variants of hOGG1 and ITGA2 polymorphisms with wild type, controlling for cigarette smoking, alcohol and salted fish consumption. Co-dominant model is used in the estimation of odds ratio. A *p*-value less than 0.05 was considered as statistically significant. Linkage disequilibrium (LD) of the 3 loci, haplotypes and their frequencies as well as association with NPC risks were determined by using web-based SNPstats software [42].

## Results

### Characteristics of study population

A total of 300 cases of histologically confirmed NPC and 533 healthy controls were available for analysis in present study. The demographic and exposure data are shown in Table 3. The average age of cases and controls was 52.8 and 53.6 years respectively. Male to female ratio for both cases and controls was 3:1. Of 300 cases, 213 (71.0%) cases were of Chinese origin, 84 (28.0%) cases were of Malay origin, and the remaining 3 (1.0%) cases were classified under other origins. Of the 533 controls, 378 (70.9%) were of Chinese origin, 150 (28.1%) controls were of Malay origin and the remaining 5 (1.0%) controls were classified under other origins.

**Table 3:** Characteristics of the study population.

Characteristics	Cases (%) N = 300	Control (%) N = 533
**Age (years)**		
**Mean (SD)**	52.8 (10.88)	53.6 (11.15)
**Gender, N (%)**		
**Male**	232 (77.3%)	407 (76.4%)
**Female**	68 (22.7%)	126 (23.6%)
**Ethnicity, N (%)**		
**Chinese**	213 (71.0%)	378 (70.9%)
**Malay**	84 (28.0%)	150 (28.1%)
**Others**	3 (1.0%)	5 (1.0%)
**Smoking status, N (%)**		
**Never**	146 (48.7%)	336 (63.0%)
**Ever**	154 (51.3%)	197 (37.0%)
**Alcohol consumption, N (%)**		
**Never**	161 (53.7%)	346 (64.9%)
**Ever**	139 (46.3%)	187 (35.1%)
**Salted Fish consumption before age of 10, N (%)**		
**Never**	103 (34.3%)	261 (49.0%)
**Ever**	197 (65.7%)	272 (51.0%)

NPC patients were more likely to ever consume salted fish at 10 years of age compared to controls (OR = 1.77, 95% CI = 1.30–2.42). Study participants with previous smoking history were more likely to develop NPC (OR = 1.81, 95% CI = 1.26–2.60). There was no significant difference observed in NPC susceptibility between study subjects who ever consumed alcohol with those who never (OR = 1.41, 95% CI = 0.97–2.06).

### Genotypic distribution of XPD Lys751Gln (rs13181), hOGG1 Ser326Cys (rs1052133) and ITGA2 C807T (rs1126643) polymorphisms

Genotypic frequencies of controls were in Hardy Weinberg Equilibrium for all 3 polymorphisms as shown in Table 4. 100% concordance was achieved between results from RFLP-PCR assay and the 10% samples sent for DNA sequencing. No significant association was observed between hOGG1 Ser326Cys and ITGA2 polymorphisms and odds of developing NPC. After adjusting for age, gender, ethnicity, cigarette smoking, alcohol consumption and salted fish consumption at age of 10 years, the OR for NPC comparing Ser/Cys and Cys/Cys genotype to wild type Ser/Ser were 1.21 (0.80–1.83) and 1.16 (0.74–1.81) respectively as shown in Table 5. For ITGA2, OR for NPC risks comparing between CT and TT genotypes to wildtype CC were 0.75 (0.54–1.03) and 0.85 (0.49–1.45) respectively (Table 5). XPD Lys751Gln polymorphism was significantly associated with NPC risk. The odds of developing NPC for genotype Lys/Lys was 1.60 (1.06–2.43) when compared to Lys/Gln and Gln/Gln as reference (Table 5).

**Table 4:** Allelic and genotypic frequencies of hOGG1 Ser326Cys (rs1052133), ITGA2 C807T (rs1126643) and XPD Lys751Gln (rs13181) polymorphism (Hardy-Weinberg Equilibrium test).

Polymorphisms		Controls (%) N = 533	X^2^ value	P value
hOGG1				
Genotypes	Ser/Ser	101 (18.9%)	0.38	0.54
	Ser/Cys	270 (50.7%)		
	Cys/Cys	162 (30.4%)		
Alleles	Ser	472 (44.3%)		
	Cys	594 (55.7%)		
ITGA2				
Genotypes	C/C	270 (50.7%)	0.30	0.58
	C/T	215 (40.3%)		
	T/T	48 (9.0%)		
Alleles	C	755 (70.8%)		
	T	311 (29.2%)		
XPD				
Genotypes	Lys/Lys	419 (78.6%)	0.19	0.66
	Lys/Gln	106 (19.9%)		
	Gln/Gln	8 (1.5%)		
Alleles	Lys	944 (88.6%)		
	Glu	122 (11.4%)		

**Table 5:** Association of polymorphisms with risk of NPC in the study population controlling for smoking, alcohol and salted fish consumption at the age of 10.

	Cases (%) N = 300	Controls (%) N = 533	B coefficient	Standard error of B	Adjusted^a^ OR^b^ (95% CI^c^)	*P* value
hOGG1 Genotypes						
Ser/Ser	50 (16.7%)	101 (18.9%)			1.00	
Ser/Cys	154 (51.3%)	270 (50.7%)	0.193	0.210	1.21 (0.80–1.83)	0.357
Cys/Cys	96 (32.0%)	162 (30.4%)	0.145	0.229	1.16 (0.74–1.81)	0.527
ITGA2 Genotypes						
C/C	173 (57.9%)	270 (50.7%)			1.00	
C/T	100 (33.4%)	215 (40.3%)	-0.292	0.167	0.75 (0.54–1.03)	0.079
T/T	26 (8.7%)	48 (9.0%)	-0.166	0.275	0.85 (0.49–1.45)	0.547
XPD Genotypes						
Lys/Lys	256 (85.3%)	419 (78.6%)	0.473	0.213	1.60 (1.06–2.43)	0.026
Lys/Gln + Gln/Gln	44 (14.6%)	114 (21.4%)			1	
Smoking status						
Never	146 (48.7%)	336 (63.0%)			1.00	
Ever	154 (51.3%)	197 (37.0%)	0.595	0.184	1.81 (1.26–2.60)	0.001
Alcohol consumption						
Never	161 (53.7%)	346 (64.9%)			1.00	
Ever	139 (46.3%)	187 (35.1%)	0.347	0.192	1.41 (0.97–2.06)	0.071
Salted Fish consumption before age of 10						
Never	103 (34.3%)	261 (49.0%)			1.00	
Ever	197 (65.7%)	272 (51.0%)	0.573	0.159	1.77 (1.30–2.42)	0.001

^a^ OR adjusted for age, gender, ethnicity, cigarette smoking, alcohol and salted fish consumption before age of 10.

^b^ OR: odds ratio

^c^ CI: confidence interval

### Linkage disequilibrium and haplotype analysis of XPD Lys751Gln (rs13181), hOGG1 Ser326Cys (rs1052133) and ITGA2 C807T (rs1126643) polymorphisms

As it is shown in Table 6, none of the aforementioned polymorphisms were observed to be non-randomly co-inherited. Calculated D’ value between polymorphisms was close to 0 which suggested that these polymorphisms were co-inherited as a random and non-selective event. Frequencies of different combinations of haplotypes in NPC cases and controls are given in Table 7. Haplotype CGC was observed to be significantly associated with NPC risk (OR = 2.20, 95% CI = 1.06–4.58) using the most frequent allele combination AGC as reference after adjustment for age, gender, ethnicity, cigarette smoking, alcohol intake and salted fish consumption at age of 10.

**Table 6:** Linkage disequilibrium between hOGG1 Ser326Cys, ITGA2 C807T and XPD Lys751Glu polymorphisms (D’).

	hOGG1 Ser326Cys	ITGA2 C807T	XPD Lys751Glu
hOGG1 Ser326Cys	-	-	-
ITGA2 C807T	0.084	-	-
XPD Lys751Glu	0.011	0.081	-

**Table 7:** Frequency distribution of haplotypes in NPC cases and controls and association with NPC risk.

Haplotype (XPD Lys751Glu /hOGG1 Ser326Cys /ITGA2 C807T)	Case Frequency	Control Frequency	Adjusted^a^ OR^b^ (95% CI^c^)	p-value
AGC	0.423	0.349	1	-
ACC	0.271	0.279	1.12 (0.83–1.52)	0.460
AGT	0.116	0.145	1.33 (0.88–2.02)	0.180
ACT	0.115	0.114	1.24 (0.83–1.84)	0.290
CGC*	0.030	0.062	2.20 (1.06–4.58)	0.035
CCC	0.022	0.031	1.99 (0.82–4.83)	0.130
CCT	0.015	0.017	0.95 (0.27–3.39)	0.940
CGT	0.009	0.004	1.33 (0.11–16.33)	0.820

^a^ OR adjusted for age, gender, ethnicity, cigarette smoking, alcohol and salted fish consumption before age of 10.

^b^ OR: odds ratio

^c^ CI: confidence interval

*: Significant value (p<0.05)

Global haplotype association p value = 0.15

## Discussion

Cigarette smoking and salted fish consumption at age of 10 were associated with increased odds of NPC. Other studies reported similar results [43–44] and hence, our results further corroborate the presumptive causal role of these environmental factors in NPC carcinogenesis. Independently of genetic factors, these habit and environmental factors (cigarette smoking and salted fish consumption) produced higher odds ratios than the SNPs indicating that the contribution of these factors to the risk of NPC could be greater than genetic factors; this is also reflected in the logit model (Table 5). We found no synergistic effect between the genotypes and environmental factors, only an additive effect. Cigarette smoking-related carcinogens including polycyclic aromatic hydrocarbons (PAH) and N-nitrosamines have been shown to cause bulky DNA adducts [45]. Failure in removing the aforementioned carcinogens from the body is postulated to increase risk of developing various cancers, namely lung, colorectal and oesophageal cancers [46–48].

The genotype distributions of hOGG1 Ser326Cys polymorphism in our study are similar to those reported by Wu et al [49]. Ser/Ser, Ser/Cys and Cys/Cys frequencies in our controls were 18.9%, 50.7% and 30.4% respectively compared with 16.0%, 49.0% and 35.0%, respectively from the Chinese study. For ITGA2, frequency of CC, CT and TT reported by Chen et al [50] were 50.7%, 40.3% and 9.0% respectively compared to 52.5%, 39.0% and 8.5% in our series. However, no significant relationship was found between both hOGG1 Ser326Cys and ITGA2 C807T polymorphisms with odds of NPC. A significant association was found between XPD Lys751Gln polymorphism and the odds of NPC. The effect of this individual polymorphism has been discussed extensively in our previous publication [28].

Haplotype analysis using SNPStats software revealed that allele combination CGC (XPD-Gln^751^/hOGG1-Cys^326^/ITGA2-C^807^) (OR = 2.20, 95% CI = 1.06–4.58) conferred higher risk of NPC using haplotype AGC as reference. The main difference between the haplotype CGC and AGC (reference) is the XPD-Gln^751^ allele. Previous studies have indicated that XPD-Gln^751^ variant was associated with several cancers, namely chronic myeloid leukaemia (CML) [51], oesophageal squamous cell carcinoma [52], digestive tract cancer [52], and hepatocellular carcinoma [53]. Apart from conferring cancer risk by itself, XPD-Gln^751^ allele was also shown in the other studies that it increases cancer risk in combination with other DNA repair genes. For example, Zhou et al have shown a significantly increased lung cancer risk in subjects carrying at least 5 variant alleles of XPD Asp312Asn, Lys751Gln and XRCC1 Arg399Gln polymorphisms compared to subjects with no variant allele [54]. In another study, Chen et al reported that increased lung cancer risk was observed in patients carrying variant alleles for both XPD Lys751Gln and XRCC1 Arg194Trp compared to patients with only 1 variant allele in the Chinese population [55]. Besides conferring higher cancer risk, XPD Lys751Gln polymorphism was shown in past studies to be associated with p53 gene mutation [55–56]. Mechanic et al found an interaction between the XPD variant alleles (Asn312 and Gln751) and the TP53 Pro72 allele for TP53 mutations [57]. XPD is a component of p53-mediated apoptosis pathway and both proteins interact directly via CTD region of XPD, where Lys751Gln is located [58]. Fibroblasts from patients with germ-line XPD mutation produce attenuated p53-mediated apoptosis, further substantiating the role of XPD in mediating cell death [59]. On the other hand, a higher risk of p53 mutation was observed in subjects with APE1 Asp/Asp plus hOGG1-Cys^326^ than in those carrying APE1-Glu plus hOGG1 Ser/Ser (OR = 3.72; 95% CI = 1.33–10.40) [60]. Given the fact that p53 tumor suppressor gene encodes for an important protein that induces growth arrest, DNA repair or cell death in response to DNA damage [61–62], inhibition of the p53 protein via mutation is an important event in early onset of carcinogenesis. Various DNA tumor viruses encode transforming oncoproteins that interact with p53 and initiate carcinogenesis through inhibition of p53-dependent programmed cell death [63]. Cells lacking functional p53 protein showed defective repair of UV damage [64]. Research using host cell reactivation (HCR) assay reported that cells with wild-type p53 showed a 3-fold higher reactivation level compared to its mutant counterpart [65].

Expression of α2β1 integrin on the platelet surface is lower in subjects carrying 807C compared to 807T allele of ITGA2 [66]. Results from an in-vitro study reported that α2 null tumor cells demonstrated enhanced anchorage-independent growth [35]. Re-expression of α2β1 in tumor cells has been reported to exhibit inhibitory effect on anchorage-independent growth of these tumor cells [67]. We postulate that the amino acid change from lysine to glutamine in XPD codon 751 decreases binding between p53 and XPD protein resulting in attenuated p53-mediated apoptosis, and hence, increasing chances of immortality for DNA damaged cells [68]. In addition, hOGG1-Cys^326^ allele tends to increase rate of p53 mutation [60] leading to a deficiency in p53-dependent apoptosis and DNA repair. Loss of α2 integrin expression due to the base change from C to T in codon 807 [66] might allow β1 integrin to contribute to cancer development through its binding with other α integrins, namely α5β1 in particular. Ligation of α5β1 to fibronectin was shown to constantly suppress apoptosis in an in-vitro study [69] and increased expression of proto-oncogene Bcl-2 (cell death antagonist) was observed as a result of integrin α5β1 ligation [70]. Therefore, effect of the interaction among 3 genes namely XPD-Gln^751^, hOGG1-Cys^326^ and ITGA2-C^807^ (haplotype CGC) could be synergistic. Attenuated p53-dependent apoptosis and upregulation of proto-oncogene Bcl-2 resulting from the aforementioned interaction could be the key to the increased NPC risk.

Although the present results may be applicable only to the Malaysian population, Malaysia—in particular peninsula Malaysia where the study was done—has a representation of 3 major ethnic groups with Malays forming the majority, comprising 68.6% of the population, followed by Chinese (23.4%) and Indians (7.0%) [71]. With different genetic pools in the study sample reflecting some of the major ethnic groups in the Malay Archipelago and the Asian continent, this study population constitutes an appropriate population for molecular epidemiological association studies. In addition, Malaysia has a sizeable minority population of Chinese origin. Given the higher NPC susceptibility of individuals of Chinese origin, and the fact that the incidence of NPC among male Malaysian Chinese is among the highest globally, results from the current study constitute a major contribution to the knowledge pool on NPC. Hence, if hOGG1 Ser326Cys, ITGA2 C807T and XPD Lys751Gln polymorphisms from the current study are verified to be valid diagnostic markers for NPC patients, the possibility exists for customizing screening modalities for high risk individuals, such as those with a family history of NPC. Since only 3% of NPC patients carry this high risk haplotype block CGC the application of hOGG1 Ser326Cys, ITGA2 C807T and XPD Lys751Gln as a diagnostic marker in a mass preventive screening program appears to be unfeasible. However, if other researchers can replicate and validate findings from the present study, the haplotype block CGC could potentially be clinically useful as a supplementary test for targeted high risk populations.

In conclusion, the allele combination CGC was significantly associated with NPC risk. Interactions between the 3 polymorphisms need to be further investigated to provide evidence for a potentiating effect among them. Other genes in the BER and NER mechanisms involved in cancer initiation should be studied to better understand NPC carcinogenesis.

## Supporting information

S1 Appendix Algorithm predicting the risk of NPC including both the environmental and genetic variables. (DOCX)

S1 Table Tables of logistic regression output involving analyses on the risk of NPC in different strata (smoking and non-smoking). (DOCX)

S2 Table Tables of analyses involving three genetic models (dominant, recessive and additive) for the association of hOGG1 Ser326Cys and ITGA2 C807T on the risk of NPC. (DOCX)

S1 Text Justifications on the selection of three specific genetic markers (hOGG1 Ser326Cys, ITGA2 C807T & XPD Lys751Gln). (DOCX)
