# Supplementary material for: Haplotype CGC from XPD, hOGG1 and ITGA2 polymorphisms increases the risk of nasopharyngeal carcinoma in Malaysia
Source: PLoS One. 2017 Nov 9;12(11):e0187200. doi: 10.1371/journal.pone.0187200 (PMC5679532; doi:10.1371/journal.pone.0187200)
Supplement: S1 Appendix — (DOCX) [file pone.0187200.s001.docx]

Appendix: Algorithm predicting the risk of NPC and prognosticating survival, depicting both environmental and genetic variables.


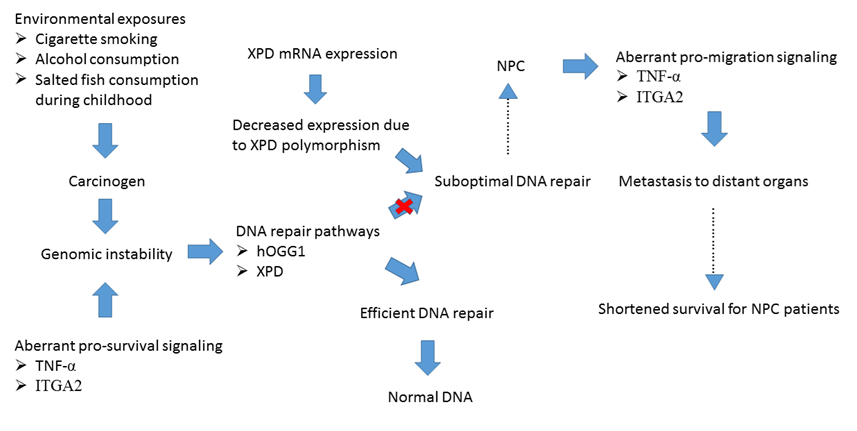


a

^a^**X** – Improper DNA repair
