# Supplementary material for: Haplotype CGC from XPD, hOGG1 and ITGA2 polymorphisms increases the risk of nasopharyngeal carcinoma in Malaysia
Source: PLoS One. 2017 Nov 9;12(11):e0187200. doi: 10.1371/journal.pone.0187200 (PMC5679532; doi:10.1371/journal.pone.0187200)
Supplement: S1 Table — (DOCX) [file pone.0187200.s002.docx]

Table A: Logistic regression: stratification by cigarette smoking, the data is analysed in the non-smoker stratum.

| **Variables in the Equation** | | | | | | | | | |
| --- | --- | --- | --- | --- | --- | --- | --- | --- | --- |
|  | | B | S.E. | Wald | df | Sig. | Exp(B) | 95% C.I.for EXP(B) | |
|  |  |  |  |  |  |  |  | Lower | Upper |
|  | hOGG1 |  |  | .190 | 2 | .910 |  |  |  |
|  | hOGG1(1)^a^ | .027 | .271 | .010 | 1 | .919 | 1.028 | .605 | 1.747 |
|  | hOGG1(2)^b^ | .116 | .301 | .148 | 1 | .701 | 1.123 | .622 | 2.025 |
|  | ITGA2 |  |  | 2.247 | 2 | .325 |  |  |  |
|  | ITGA2(1)^c^ | -.239 | .216 | 1.224 | 1 | .269 | .787 | .515 | 1.203 |
|  | ITGA2(2)^d^ | -.478 | .391 | 1.495 | 1 | .221 | .620 | .288 | 1.334 |
|  | XPD(1)^e^ | .407 | .268 | 2.306 | 1 | .129 | 1.503 | .888 | 2.542 |
|  | SaltedFish(1) | .767 | .210 | 13.361 | 1 | .000 | 2.153 | 1.427 | 3.248 |
|  | Alcohol(1) | .054 | .231 | .054 | 1 | .816 | 1.055 | .671 | 1.659 |
|  | Constant | -1.541 | .368 | 17.517 | 1 | .000 | .214 |  |  |
| ^a^hOGG1 Ser/Cys genotype  ^b^hOGG1 Cys/Cys genotype  ^c^ITGA2 C/T genotype  ^d^ITGA2 T/T genotype  ^e^XPD Lys/Lys genotype | | | | | | | | | |

Table B: Logistic regression: stratification by cigarette smoking, the data is analysed in the smoker stratum.

| **Variables in the Equation** | | | | | | | | | |
| --- | --- | --- | --- | --- | --- | --- | --- | --- | --- |
|  | | B | S.E. | Wald | df | Sig. | Exp(B) | 95% C.I.for EXP(B) | |
|  |  |  |  |  |  |  |  | Lower | Upper |
|  | hOGG1 |  |  | 1.047 | 2 | .592 |  |  |  |
|  | hOGG1(1)^a^ | .303 | .317 | .917 | 1 | .338 | 1.354 | .728 | 2.520 |
|  | hOGG1(2)^b^ | .145 | .335 | .187 | 1 | .665 | 1.156 | .599 | 2.229 |
|  | ITGA2 |  |  | 3.781 | 2 | .151 |  |  |  |
|  | ITGA2(1)^c^ | -.425 | .239 | 3.158 | 1 | .076 | .654 | .409 | 1.045 |
|  | ITGA2(2)^d^ | .122 | .391 | .097 | 1 | .756 | 1.129 | .525 | 2.429 |
|  | XPD(1)^e^ | .525 | .301 | 3.042 | 1 | .081 | 1.690 | .937 | 3.049 |
|  | SaltedFish(1) | .410 | .226 | 3.289 | 1 | .070 | 1.506 | .967 | 2.345 |
|  | Alcohol(1) | .387 | .228 | 2.890 | 1 | .089 | 1.472 | .943 | 2.300 |
|  | Constant | -1.201 | .417 | 8.302 | 1 | .004 | .301 |  |  |
| ^a^hOGG1 Ser/Cys genotype  ^b^hOGG1 Cys/Cys genotype  ^c^ITGA2 C/T genotype  ^d^ITGA2 T/T genotype  ^e^XPD Lys/Lys genotype | | | | | | | | | |
