# Supplementary material for: Haplotype CGC from XPD, hOGG1 and ITGA2 polymorphisms increases the risk of nasopharyngeal carcinoma in Malaysia
Source: PLoS One. 2017 Nov 9;12(11):e0187200. doi: 10.1371/journal.pone.0187200 (PMC5679532; doi:10.1371/journal.pone.0187200)
Supplement: S2 Table — (DOCX) [file pone.0187200.s003.docx]

Table A: Logistic regression: hOGG1 polymorphism was analysed using the dominant, recessive and additive models.

| **Model** | **Genotype** | **Control** | **Case** | **OR (95% CI)** | **P-value** |
| --- | --- | --- | --- | --- | --- |
| Dominant | G/G | 162 (30.4%) | 96 (32%) | 1.00 | 0.82 |
|  | C/G-C/C | 371 (69.6%) | 204 (68%) | 0.96 (0.71-1.32) |  |
| Recessive | G/G-C/G | 432 (81%) | 250 (83.3%) | 1.00 | 0.46 |
|  | C/C | 101 (18.9%) | 50 (16.7%) | 0.87 (0.59-1.27) |  |
| Log-additive | --- | --- | --- | 0.94 (0.76-1.16) | 0.57 |

Table B: Logistic regression: ITGA2 polymorphism was analysed using the dominant, recessive and additive models.

| **Model** | **Genotype** | **Control** | **Case** | **OR (95% CI)** | **P-value** |
| --- | --- | --- | --- | --- | --- |
| Dominant | C/C | 270 (50.7%) | 171 (57%) | 1.00 | 0.06 |
|  | C/T-T/T | 263 (49.3%) | 129 (43%) | 0.75 (0.56-1.01) |  |
| Recessive | C/C-C/T | 485 (91%) | 273 (91%) | 1.00 | 0.96 |
|  | T/T | 48 (9%) | 27 (9%) | 0.99 (0.60-1.64) |  |
| Log-additive | --- | --- | --- | 0.84 (0.67-1.06) | 0.14 |
