# Supplementary material for: Haplotype CGC from XPD, hOGG1 and ITGA2 polymorphisms increases the risk of nasopharyngeal carcinoma in Malaysia
Source: PLoS One. 2017 Nov 9;12(11):e0187200. doi: 10.1371/journal.pone.0187200 (PMC5679532; doi:10.1371/journal.pone.0187200)
Supplement: S1 Text — (DOCX) [file pone.0187200.s004.docx]

hOGG1 Ser326Cys:

For lung cancer, homozygous Cys/Cys genotype was significantly associated with 2-fold increase in cancer risk when compared to Ser/Ser genotype (OR=2.1, 95% CI=1.2-3.7) (Le Marchand et al., 2002). Similar results were observed in another lung cancer study where subjects with Cys/Cys genotype were at an increased risk of squamous cell carcinoma and adenocarcinoma compared to those with Ser/Cys and Ser/Ser genotypes (OR=2.18, 95% CI=1.05-4.54) (Sugimura et al., 1999). As for orolaryngeal cancer, hOGG1 326 Ser/Cys and Cys/Cys genotypes were both observed with a significant increased risk of cancer with the odds ratio of 1.6 (95% CI=1.04-2.60) and 4.1 (95% CI=1.3-13), respectively (Elahi et al., 2002). hOGG1 Ser326Cys polymorphism was also reported to be significantly associated with increased risk of colorectal cancer, with Ser/Cys + Cys/Cys genotypes having 1.4-times (95% CI=1.03-1.85) higher risk as compared to Ser/Ser genotype (Lai et al., 2016). Significant results were also observed in head and neck cancer where individuals carrying Ser326Cys and Cys326Cys were at an increased risk for head and neck squamous cell carcinoma (HNSCC) with the respective odds ratio of 1.69 (95% CI=1.19-2.45) and 4.56 (95% CI=2.07-10.05) (Sliwinski et al., 2011). A similar pattern of association has been shown by Cho et al (2003) and Laantri et al (2011) where increased risk of NPC was found in individuals carrying Ser326Cys and Cys326Cys (OR= 1.6, 95% CI= 1.0-2.6) and in individuals with Cys326Cys alone (OR= 1.22, 95% CI= 0.77-1.90).

ITGA2 C807T:

A significant association was found between the ITGA2 C807T polymorphism and gastric cancer risk. Individuals with variant genotypes (C/T + T/T) had a higher risk of gastric cancer (OR=1.57, 95% CI=1.13-2.17) compared to those with CC genotype (Chen *et al.*, 2011). Similar results were observed in a breast cancer study where individuals carrying 807 C/C genotype had a significantly lower risk of cancer compared to those with variant genotypes (OR=0.72, 95% CI=0.53-0.98) ([Langsenlehner *et al.*, 2006](#_ENREF_279)).

XPD Lys751Gln:

G allele of XPD Lys751Gln polymorphism was associated with increased risk of prostate cancer, according to Yang and colleagues (2013) (OR=1.53, 95% CI= 1.04-2.37). Heterozygous genotype of XPD Lys751Gln polymorphism has been associated with increased breast cancer risk (OR=4.74, 95% CI=9.03-9.92) (Hosseini et al., 2009). Homozygous variant genotype of XPD Lys751Gln polymorphism increased the risk for chronic myeloid leukemia (OR=2.37, 95% CI=1.20-4.67) (Banescu et al., 2014) while heterozygous and homozygous variant genotypes combined increased risk of acute myeloid leukemia (OR=2.55, 95% CI=1.53-4.25) (Banescu et al., 2014). Homozygous variant genotype of XPD was found to be significantly associated with increased risk of acute lymphoblastic leukemia (OR=1.83, 95%CI=1.21-2.75) (Wang et al., 2011). Similarly, XPD Lys751Gln polymorphism was shown to increase risk of oral SCC in women carrying Lys/Gln genotypes compared to those with Lys/Lys genotype (OR=3.43, 95% CI= 1.40-8.37) (Farnebo et al., 2015). An increased risk for digestive tract cancer was found among those with homozygous variant genotype of XPD Lys751Gln (Huang et al., 2012).

References:

1. Le Marchand, L., Donlon, T., Lum-Jones, A., Seifried, A., & Wilkens, L. R. (2002). Association of the hOGG1 Ser326Cys polymorphism with lung cancer risk. *Cancer Epidemiol Biomarkers Prev, 11*(4), 409-412.
2. Sugimura, H., Kohno, T., Wakai, K., Nagura, K., Genka, K., Igarashi, H., Morris, B. J., Baba, S., Ohno, Y., Gao, C., Li, Z., Wang, J., Takezaki, T., Tajima, K., Varga, T., Sawaguchi, T., Lum, J. K., Martinson, J. J., Tsugane, S., Iwamasa, T., Shinmura, K., & Yokota, J. (1999). hOGG1 Ser326Cys polymorphism and lung cancer susceptibility. *Cancer Epidemiol Biomarkers Prev, 8*(8), 669-674.
3. Elahi, A., Zheng, Z., Park, J., Eyring, K., McCaffrey, T., & Lazarus, P. (2002). The human OGG1 DNA repair enzyme and its association with orolaryngeal cancer risk. *Carcinogenesis, 23*(7), 1229-1234.
4. Lai, C. Y., Hsieh, L. L., Tang, R., Santella, R. M., Chang-Chieh, C. R., & Yeh, C. C. (2016). Association between polymorphisms of APE1 and OGG1 and risk of colorectal cancer in Taiwan. *World J Gastroenterol, 22*(12), 3372-3380. doi: 10.3748/wjg.v22.i12.3372.
5. Sliwinski, T., Przybylowska, K., Markiewicz, L., Rusin, P., Pietruszewska, W., Zelinska-Blizniewska, H., Olszewski, J., Morawiec-Sztandera, A., Mlynarski, W., & Majsterek, I. (2011). MUTYH Tyr165Cys, OGG1 Ser326Cys and XPD Lys751Gln polymorphisms and head neck cancer susceptibility: a case control study. *Mol Biol Rep, 38*(2), 1251-1261. doi: 10.1007/s11033-010-0224-x.
6. Laantri, N., Jalbout, M., Khyatti, M., Ayoub, W. B., Dahmoul, S., Ayad, M., Bedadra, W., Abdoun, M., Mesli, S., Kandil, M., Hamdi-Cherif, M., Boualga, K., Bouaouina, N., Chouchane, L., Benider, A., Ben-Ayed, F., Goldgar, D., & Corbex, M. (2011). XRCC1 and hOGG1 genes and risk of nasopharyngeal carcinoma in North African countries. *Mol Carcinog, 50*(9), 732-737. doi: 10.1002/mc.20754.
7. Cho, E. Y., Hildesheim, A., Chen, C. J., Hsu, M. M., Chen, I. H., Mittl, B. F., Levine, P. H., Liu, M. Y., Chen, J. Y., Brinton, L. A., Cheng, Y. J., & Yang, C. S. (2003). Nasopharyngeal carcinoma and genetic polymorphisms of DNA repair enzymes XRCC1 and hOGG1. *Cancer Epidemiol Biomarkers Prev, 12*(10), 1100-1104.
8. Chen, J., Liu, N. N., Li, J. Q., Yang, L., Zeng, Y., Zhao, X. M., Xu, L. L., Luo, X., Wang, B., & Wang, X. R. (2011). Association between ITGA2 C807T polymorphism and gastric cancer risk. *World J Gastroenterol, 17*(23), 2860-2866. doi: 10.3748/wjg.v17.i23.2860.
9. Langsenlehner, U., Renner, W., Yazdani-Biuki, B., Eder, T., Wascher, T. C., Paulweber, B., Clar, H., Hofmann, G., Samonigg, H., & Krippl, P. (2006). Integrin alpha-2 and beta-3 gene polymorphisms and breast cancer risk. *Breast Cancer Res Treat, 97*(1), 67-72. doi: 10.1007/s10549-005-9089-4.
10. Hosseini, M., Houshmand, M., & Ebrahimi, A. (2009). The ERCC2 K751 polymorphism is associated with breast cancer risk. *Archives of Medical Science, 5*(3), 456-458.
11. Yang, B., Chen, W. H., Wen, X. F., Liu, H., & Liu, F. (2013). Role of DNA repair-related gene polymorphisms in susceptibility to risk of prostate cancer. *Asian Pac J Cancer Prev, 14*(10), 5839-5842.
12. Banescu, C., Trifa, A. P., Demian, S., Benedek Lazar, E., Dima, D., Duicu, C., & Dobreanu, M. (2014). Polymorphism of XRCC1, XRCC3, and XPD genes and risk of chronic myeloid leukemia. *Biomed Res Int, 2014*, 213790. doi: 10.1155/2014/213790
13. Wang, Y. H., Yeh, S. D., Shen, K. H., Shen, C. H., Tung, M. C., Liu, C. T., & Chiou, H. Y. (2011). Association of hOGG1 and XPD polymorphisms with urothelial carcinoma in Taiwan. *Anticancer Res, 31*(11), 3939-3944.
14. Farnebo, L., Stjernstrom, A., Fredrikson, M., Ansell, A., Garvin, S., & Thunell, L. K. (2015). DNA repair genes XPC, XPD, XRCC1, and XRCC3 are associated with risk and survival of squamous cell carcinoma of the head and neck. *DNA Repair (Amst), 31*, 64-72. doi: 10.1016/j.dnarep.2015.05.003.
15. Huang, C. G., Liu, T., Lv, G. D., Liu, Q., Feng, J. G., & Lu, X. M. (2012). Analysis of XPD genetic polymorphisms of esophageal squamous cell carcinoma in a population of Yili Prefecture, in Xinjiang, China. *Mol Biol Rep, 39*(1), 709-714. doi: 10.1007/s11033-011-0789-z.
